# Cold temperature improves mobility and survival in *Drosophila* models of autosomal-dominant hereditary spastic paraplegia (AD-HSP)

**DOI:** 10.1242/dmm.013987

**Published:** 2014-06-06

**Authors:** Sally L. Baxter, Denise E. Allard, Christopher Crowl, Nina Tang Sherwood

**Affiliations:** 1 Perelman School of Medicine at the University of Pennsylvania, Philadelphia, PA 19104, USA.; 2 Department of Biology, University of North Carolina at Chapel Hill, Chapel Hill, NC 27514, USA.; 3 Department of Biology, Duke University, Durham, NC 27708, USA.

**Keywords:** Spastin, Hereditary spastic paraplegia, AD-HSP, Microtubule severing, Cold treatment, Therapeutic hypothermia, *Drosophila* disease model

## Abstract

Autosomal-dominant hereditary spastic paraplegia (AD-HSP) is a crippling neurodegenerative disease for which effective treatment or cure remains unknown. Victims experience progressive mobility loss due to degeneration of the longest axons in the spinal cord. Over half of AD-HSP cases arise from loss-of-function mutations in *spastin*, which encodes a microtubule-severing AAA ATPase. In *Drosophila* models of AD-HSP, larvae lacking Spastin exhibit abnormal motor neuron morphology and function, and most die as pupae. Adult survivors display impaired mobility, reminiscent of the human disease. Here, we show that rearing pupae or adults at reduced temperature (18°C), compared with the standard temperature of 24°C, improves the survival and mobility of adult *spastin* mutants but leaves wild-type flies unaffected. Flies expressing human *spastin* with pathogenic mutations are similarly rescued. Additionally, larval cooling partially rescues the larval synaptic phenotype. Cooling thus alleviates known *spastin* phenotypes for each developmental stage at which it is administered and, notably, is effective even in mature adults. We find further that cold treatment rescues larval synaptic defects in flies with mutations in Flower (a protein with no known relation to Spastin) and mobility defects in flies lacking Kat60-L1, another microtubule-severing protein enriched in the CNS. Together, these data support the hypothesis that the beneficial effects of cold extend beyond specific alleviation of Spastin dysfunction, to at least a subset of cellular and behavioral neuronal defects. Mild hypothermia, a common neuroprotective technique in clinical treatment of acute anoxia, might thus hold additional promise as a therapeutic approach for AD-HSP and, potentially, for other neurodegenerative diseases.

## INTRODUCTION

Hereditary spastic paraplegias (HSPs) are a group of neurodegenerative disorders marked by lower-limb spasticity (stiffness) and weakness, leading to progressive difficulty walking (Fink, 2013; http://www.sp-foundation.org/). Supportive treatments exist, but are of mixed efficacy and do not restore mobility. The most common form, pure autosomal dominant HSP (AD-HSP), accounts for 70–80% of HSP-afflicted families. AD-HSP pathology is characterized primarily by degeneration of the longest descending axons of the central nervous system (CNS). These originate from the upper motor neurons in the cortex and terminate in the lumbar spine, innervating the α1 motor neurons that control leg movement. The relative specificity of the affected neuronal population is striking, but still not understood.

Over 50% of cases of pure AD-HSP are caused by mutations in *spastin* (Hazan et al., 1999), which encodes one of a small family of microtubule-severing proteins – hexameric ATPases that disassemble microtubules along their length (Roll-Mecak and McNally, 2010; Sharp and Ross, 2012). Although it is not yet clear why Spastin is important in neurons, one thought is that its severing activity shortens microtubules for efficient transport into axons (Errico et al., 2002; Yu et al., 2008). Other models suggest that Spastin is required for net microtubule loss (Trotta et al., 2004) or growth (Sherwood et al., 2004) at motor neuron synapses, axon guidance (Wood et al., 2006; Butler et al., 2010) and axon transport (Kasher et al., 2009; Fassier et al., 2013). Most recently, data has emerged supporting roles for Spastin in membrane regulation, promoting tubular endoplasmic reticulum (Park et al., 2010) and endosomal tubule formation (Allison et al., 2013). Much progress has been made, but Spastin’s relevant functions, regulatory pathways and the specific mechanisms by which its mutations lead to axonal degeneration remain unclear.

*Drosophila melanogaster* is an effective model system for study of a wide variety of neurodegenerative diseases because of its high conservation of neuronal gene function with humans, short generation time, well-characterized features and the availability of a wide range of genetic and experimental tools (Bilen and Bonini, 2005). In regards to providing a model for AD-HSP, *Drosophila* Spastin, like its vertebrate orthologs, severs purified microtubules and those in *Drosophila* S2 cells (Roll-Mecak and Vale, 2005). Knocking down fly Spastin using a RNA-interference (RNAi) transgene (Trotta et al., 2004) or deletion of the endogenous gene (Sherwood et al., 2004) both cause synaptic defects at the *Drosophila* larval neuromuscular junction (NMJ), supporting a role for *spastin* in regulating synaptic morphology and function. Orthologs of several other HSP causative genes studied in *Drosophila* also exhibit progressive neurodegeneration, supporting the relevance of flies in providing insights into mechanisms underlying this disease (Wang and O’Kane, 2008; Ozdowski et al., 2014).

The *spastin* gene is completely deleted in the *spastin^5.75^ Drosophila* model of AD-HSP (Sherwood et al., 2004). *Spastin^5.75^* larvae are homozygous-viable and have no obvious behavioral defects, but electrophysiological analysis at the NMJ reveals that synaptic strength is reduced. Morphological analysis reveals smaller, more numerous synaptic boutons that are often arranged in bunches and contain only sparse microtubules. Adult *spastin^5.75^* flies rarely eclose (emerge from their pupal cases) and those that do have severely reduced lifespans, neither fly nor jump, walk and climb only poorly and, even when still, hold their legs unsteadily. These phenotypes are equivalently rescued by low level, neuron-specific expression of *Drosophila* or human wild-type *spastin* transgenes, indicating that Spastin is predominantly required in neurons, and functions similarly in both organisms (Sherwood et al., 2004; Du et al., 2010).

**TRANSLATIONAL IMPACT**

**Clinical issue**

Autosomal dominant hereditary spastic paraplegia, or AD-HSP, is an inherited neurodegenerative disease that manifests as early as toddlerhood, causing progressive loss of mobility. The primary symptoms, leg spasticity and weakness, arise from localized degeneration of the longest central nervous system axons. The identification of several causative genes has made the study of AD-HSP using model systems eminently feasible, and the fruit fly *Drosophila* has been used extensively to explore the underlying pathology. *Spastin*, the gene most commonly mutated in AD-HSP, encodes a member of the microtubule-severing protein family in both humans and flies, and flies that lack *spastin* also exhibit compromised mobility. Despite significant progress since its discovery, a clear understanding of the role of Spastin in the nervous system is lacking, and there are still no reliable therapies for AD-HSP. This study explores a fortuitous observation that cold temperatures alleviate symptoms in *Drosophila* models of *spastin*-mediated AD-HSP, in order to investigate whether cooling could provide a therapeutic approach to this disease in humans.

**Results**

The authors test their hypothesis that cold treatment mitigates behavioral and cellular *spastin* mutant defects by examining eclosion (emergence of adult flies from the pupal case), mobility, lifespan and synapse morphology in mutant and wild-type flies reared in cold conditions during discrete developmental periods. They find that in *spastin* null animals, as well as in mutant flies that model AD-HSP through the expression of pathogenic human *spastin*, cooling alleviates the reduced lifespan, slowed mobility and aberrant synapse morphology caused by *spastin* loss. Cold-induced alleviation of phenotypes was most effective during the developmental periods, when spastin is required, consistent with the effects being mediated through a mechanism linked with spastin loss. The effects were not seen in wild-type animals, demonstrating specific mitigation of mutant phenotypes. The authors further show that cooling alleviates neuronal defects due to mutations in a related microtubule-severing protein, Kat-60L1, and in Flower, which regulates synaptic vesicle endocytosis. These results provide evidence that moderate hypothermia could be broadly effective in alleviating neurodegenerative phenotypes.

**Implications and future directions**

Therapeutic hypothermia is commonly employed in clinical settings to prevent anoxia-induced neurological damage following stroke and cardiac arrest, although the mechanisms for its efficacy remain unknown. This work implicates cooling as a novel therapeutic approach for neuronal dysfunction in AD-HSP, and potentially in other neurodegenerative diseases. Future studies should address the effects of localized cold treatment in vertebrate models of AD-HSP, and utilize the *Drosophila* model system to systematically investigate the underlying molecular mechanisms and spatiotemporal requirements for the neuroprotective effects of hypothermia. The work also highlights the finding that neuronal phenotypes are potentially very sensitive to variations in temperature, which should be taken into account when designing studies of neurodegenerative phenotypes in model systems.

Surprisingly, *spastin^5.75^* eclosion improves dramatically when animals are raised at a reduced temperature of 18°C, compared with the typical rearing temperature of 24–25°C. Maintaining flies at 18°C is a standard fly husbandry technique that slows development roughly twofold, but is not associated with systematic loss of phenotype or enhancement of viability and can in fact adversely affect stock health. Furthermore, although temperature-sensitive mutations are common, these typically arise from differences in mutant versus wild-type protein folding, which is impossible in *spastin^5.75^* animals given that the gene is completely deleted. This suggests that the cold-rescuing effect is specific to Spastin dysfunction, and could therefore provide a novel therapeutic approach to AD-HSP.

To quantify the effects of cooling, we determined eclosion rate, climb rate and lifespan for *spastin* null animals reared at 18°C, and compared them with values for untreated (non-temperature-shifted) mutant animals raised at 24°C, as well as with control *white-CantonS* (*WCS*) animals raised in parallel conditions. We looked at whether the temporal parameters for the cold effect made sense in the context of when *spastin* is required. We repeated these experiments using transgenic strains that express human *spastin* genes, to address whether hypothermic rescue held true for flies with partial human *spastin* function, which are more representative of the human disease. Finally, we tested the hypothesis that cold functionally substitutes for Spastin’s severing activity through the promotion of microtubule disassembly, and discovered instead that the alleviatory effects of cooling also extend to other mutations causing synaptic dysfunction. Mild hypothermia is commonly employed for neuroprotective purposes during treatment of stroke, cardiac arrest and other ischemic trauma; our data indicate that cooling might also be beneficial in AD-HSP and other neurodegenerative contexts.

## RESULTS

### Pupal stage cold treatment improves *spastin* mutant eclosion

Most *Drosophila* lacking *spastin* survive into metamorphosis but fail to emerge from the pupal case. However, *spastin^5.75^* nulls reared at 18°C rather than 24°C were considerably more successful at reaching adulthood. We quantified this cold-induced alleviation of pupal lethality and tested for a developmental period(s) during which it is effective. Cooling could be required throughout development, or alternatively, be necessary only at a particular stage. The former would support a broad effect, such as general metabolism or developmental rate. The latter, if coinciding with temporal requirements for Spastin, would support a mechanism relevant to the loss of Spastin function in the nervous system.

**Fig. 1.**
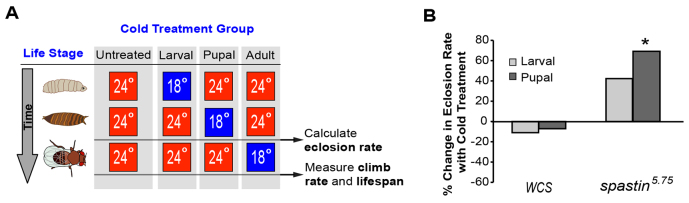
**Pupal stage cooling increases *spastin^5.75^* eclosion.** (A) Schematic of the experimental paradigms and data gathered throughout this study, for untreated (no cold treatment), larval, pupal, and adult stage cold administration. (B) Eclosion of homozygous *spastin^5.75^* null mutants increases over 70% when animals are reared at 18°C as pupae rather than exclusively at 24°C (*P*<0.02). Larval cooling might also have some positive effect, although this did not reach statistical significance (*P*>0.30). By contrast, *WCS* eclosion is unaffected by cold treatment. *P<0.05.

*White-CantonS* (*WCS*) controls and *spastin^5.75^* mutant *Drosophila* were raised in parallel at 24°C (‘untreated’) or moved to 18°C during either their larval or pupal stage of development, and differences in eclosion rates determined for each condition (Fig. 1A,B). Compared with the 86% eclosion rate of *WCS* flies, only 18% of homozygous *spastin^5.75^* flies maintained at 24°C eclosed (Table 1). Cold treatment specifically during the larval stage resulted in slightly, but not significantly, higher mutant eclosion (Table 1; Fig. 1B). Only pupal stage cold treatment produced a significant increase to levels that, although still well below wild type, were 70% greater than those for untreated mutants (Fig. 1B). This effect was specific to the *spastin* mutation, as cold did not affect *WCS* eclosion.

**Table 1.**
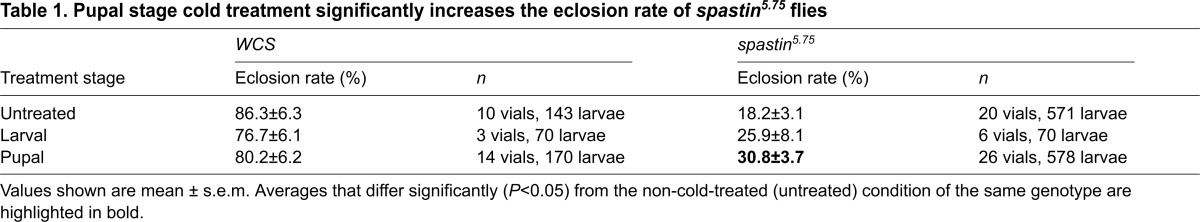
Pupal stage cold treatment significantly increases the eclosion rate of *spastin^5.75^* flies

### Both pupal and adult cold treatment improve adult mobility

**Fig. 2.**
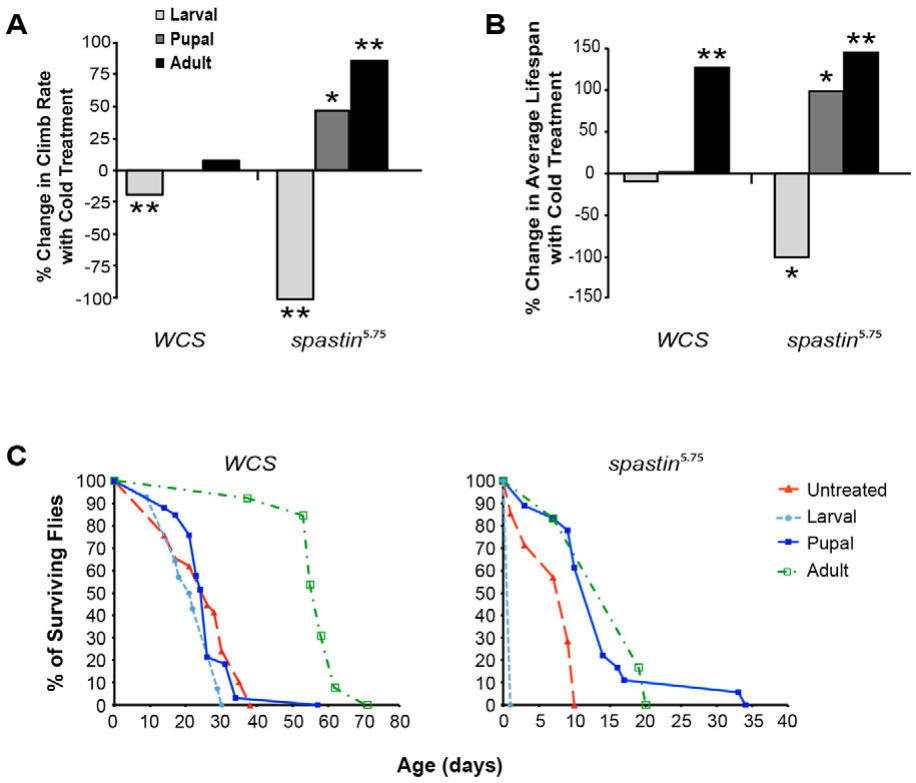
**Pupal or adult-stage cooling increases mobility and lifespan of *spastin^5.75^* flies.** (A) *Spastin^5.75^* flies cold-treated as pupae or adults climb faster than untreated mutants kept at 24°C (*P*<0.05 or *P*<6×10^−3^, respectively). Cold has no effect on *WCS* mobility except when administered during the larval stage, which decreases climb rates by almost 20% (*P*<3×10^−3^). Larval cooling is even more deleterious to mutants, which are unable to climb after eclosion. (B) Average adult mutant lifespan is also considerably reduced by larval cooling, but is nearly doubled by pupal cooling (*P*<0.05). Average *WCS* lifespan is affected only by adult-stage cold treatment, which affects mutants to a similar degree (*P*<2×10^−3^). (C) Left: Survival curves overlap between untreated *WCS* flies (red triangles, long dashed line) and *WCS* flies cooled as larvae (light blue circles, short dashed line) or pupae (dark blue squares, solid line). Adult stage cooling (green boxes, dotted dashed line) right-shifts the population as a whole, doubling average lifespan. Right: The population of cold-treated *spastin^5.75^* adults is similarly right-shifted relative to untreated mutants. However, pupal cooling also right-shifts the *spastin^5.75^* survival curve and extends maximum lifespan over threefold compared with untreated mutants. *P<0.05; **P<0.005.

We next looked at whether cold also improves the dramatically weakened mobility of *spastin* mutant flies, by measuring adult climbing rates (Table 2; Fig. 2A). All mutants that eclosed following larval stage cold treatment did not climb, dying shortly after eclosion. However, *spastin^5.75^* flies cold-treated as pupae climbed 46% faster than mutants maintained at 24°C, and when cold-treated as adults, climbed 85% faster. By contrast, *WCS* climbing was unaffected by pupal and adult cold treatment, and also impaired by larval-stage treatment (Fig. 2A).

**Table 2.**
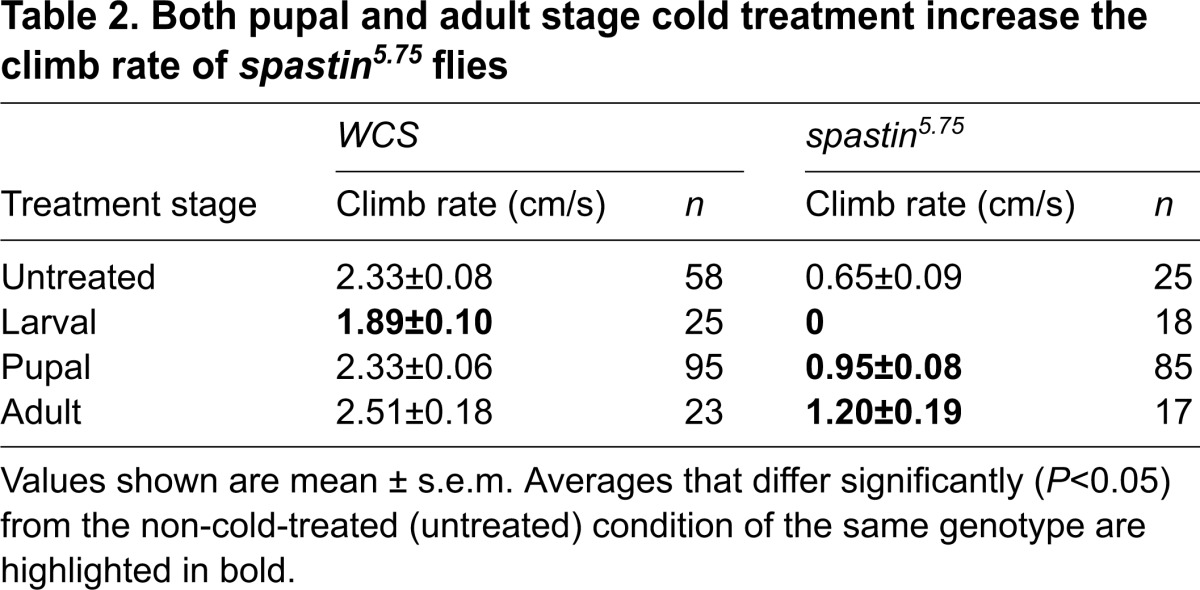
Both pupal and adult stage cold treatment increase the climb rate of *spastin^5.75^* flies

### Pupal stage cold treatment extends lifespan

Besides compromised mobility, *spastin^5.75^* flies are short-lived compared with wild type (Sherwood et al., 2004). Whereas *WCS* flies at 24°C lived 3–4 weeks on average, mutants survived only 1 week (Fig. 2B,C, Table 3). As in the climb rate experiments, larval cold treatment was deleterious to mutant fly survival. Adult-stage cold treatment extended the average lifespan 2.5-fold but this was not different from *WCS* flies, which lived 2.3-fold longer when cold-treated as adults (Fig. 2B,C). However, whereas pupal cold treatment left *WCS* flies unaffected, *spastin^5.75^* flies lived twice as long as untreated mutants (Fig. 2B). The survival curve for pupal cold-treated *spastin^5.75^* flies (dark blue solid line) showed a clear right shift of the entire population relative to untreated mutant flies (red long dashed line; Fig. 2C). Notably, about 10% of treated mutants had considerably extended lifetimes, which were threefold longer than untreated mutants and comparable to the maximal lifespan of control *WCS* flies.

**Table 3.**
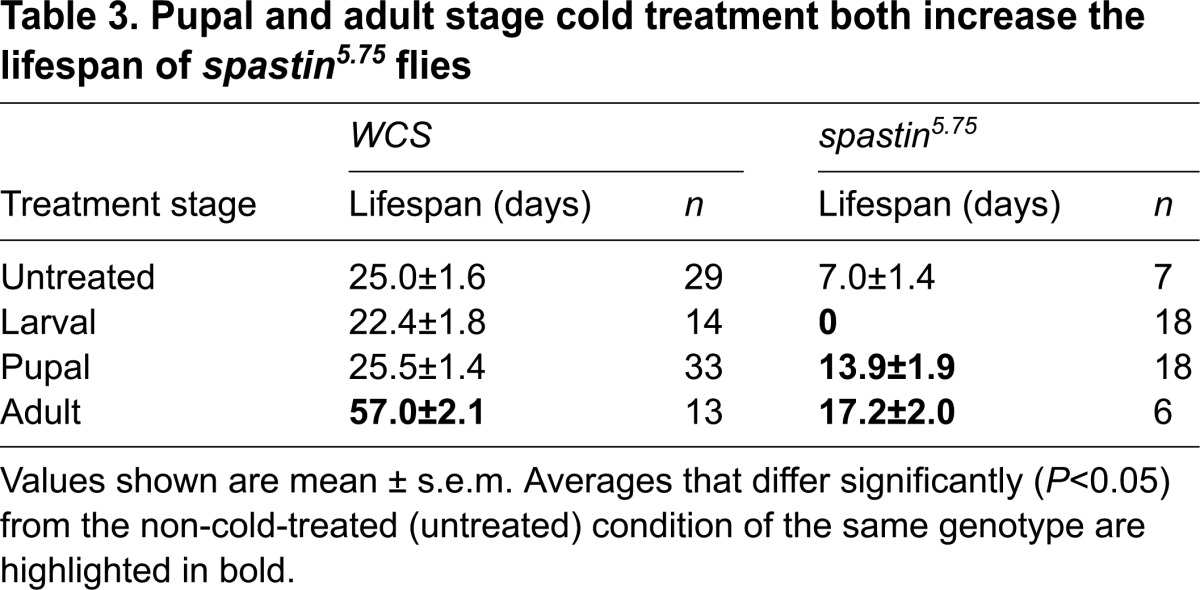
Pupal and adult stage cold treatment both increase the lifespan of *spastin^5.75^* flies

These measurements indicate that pupal development is an effective period for cold to enhance eclosion, mobility and lifespan of adult *spastin^5.75^* mutants, although it has little effect on wild-type flies. Additionally, cooling significantly improves mobility even when applied in *spastin^5.75^* adults, when the nervous system has already matured. Larval cooling, by contrast, exacerbates *spastin* mutant phenotypes in adults. Taken together, these data indicate that cooling-induced rescue of adult phenotypes arises from the effects of mild hypothermia on the adult nervous system, which is assembled *de novo* during pupal stages as part of metamorphosis.

### Pupal stage cold treatment improves eclosion and mobility of AD-HSP genotype flies

We sought further evidence for the specificity of cold rescue to loss of Spastin function by performing the previous experiments in a *Drosophila* AD-HSP model that recapitulates *spastin* gene dosage and allelic severity of the human disease (Du et al., 2010). We used two fly strains designed to genocopy humans at the *spastin* locus. In the first, denoted *H^WT^,H^WT^*, flies lacking endogenous *spastin* (i.e. *spastin^5.75^* flies) instead express wild-type human Spastin, encoded by two copies of a human *spastin* transgene, in all neurons. The second genotype, *H^L44^,H^R388^*, mimics the most severe form of *spastin*-mediated AD-HSP in humans (Svenson et al., 2004). Instead of wild-type human *spastin*, these flies carry one copy each of transgenes encoding the S44L or K388R human Spastin mutations in the fly null background. Pan-neuronal *H^WT^,H^WT^* expression rescues *spastin*-null phenotypes equally as well as wild-type *Drosophila spastin* transgenes, demonstrating functional conservation between human and fly Spastin. Flies neuronally expressing *H^L44^,H^R388^*, however, are severely compromised in eclosion, mobility and survival. Thus, the *H^WT^,H^WT^* flies provided a genetic control group (because, like *WCS*, these express wild-type *spastin*) and the *H^L44^,H^R388^* flies modeled severe AD-HSP.

**Fig. 3.**
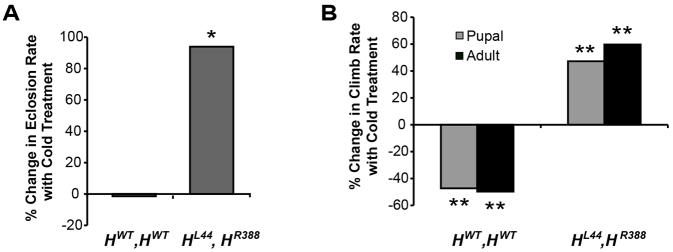
**Pupal stage cooling increases eclosion and climb rate in ‘AD-HSP’ flies.** (A) Nearly 100% more animals expressing mutant human *spastin* (genotype *H^L44^,H^R388^*) eclose if cooled as pupae, compared with those maintained at 24°C (*P*<0.03), whereas eclosion of flies expressing wild-type human *spastin* (*H^WT^,H^WT^*) is unaffected by cold. (B) *H^L44^,H^R388^* flies cold-treated as pupae or adults climb nearly 50% (*P*<2×10^−3^) and 60% (*P*<6×10^−4^) faster, respectively. By contrast, cooling decreases climb rates of *H^WT^,H^WT^* flies by 50% for both pupal (*P*<1×10^−4^) and adult treatments (*P*<7×10^−4^). *P<0.05; **P<0.005.

As further evidence of the therapeutic effect of cold, *H^L44^,H^R388^* flies cold-treated as pupae eclosed nearly twice as often as those kept at 24°C, whereas *H^WT^,H^WT^* eclosion was unaffected by cold (Fig. 3A; Table 4). *H^L44^,H^R388^* flies also climbed 47% and 60% faster, respectively, if cold-treated as pupae or adults (Fig. 3B, Table 5), but cold treatment of *H^WT^,H^WT^* at these stages yielded adults that climbed only half as quickly. Although *spastin* transgene expression might have been reduced at 18°C, our previous studies showed that a single copy of *H^WT^* rescues the null mutants as effectively as two copies, making it unlikely that temperature-based alterations in expression could account for the alleviatory effects seen here (Du et al., 2010). Overall, the parallels between these results and those for *spastin^5.75^* strongly support the specificity of cold alleviation to defects caused by Spastin dysfunction in the nervous system.

**Table 4.**
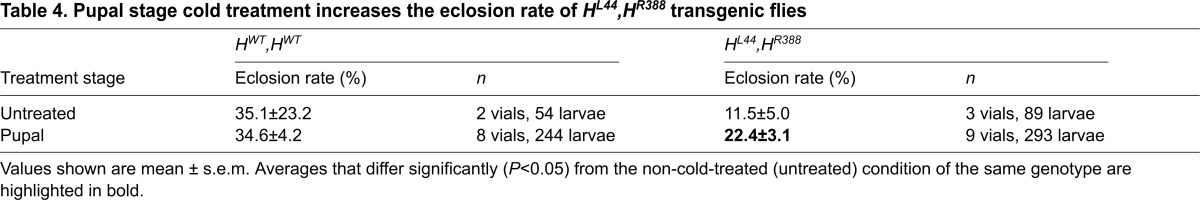
Pupal stage cold treatment increases the eclosion rate of *H^L44^,H^R388^* transgenic flies

**Table 5.**
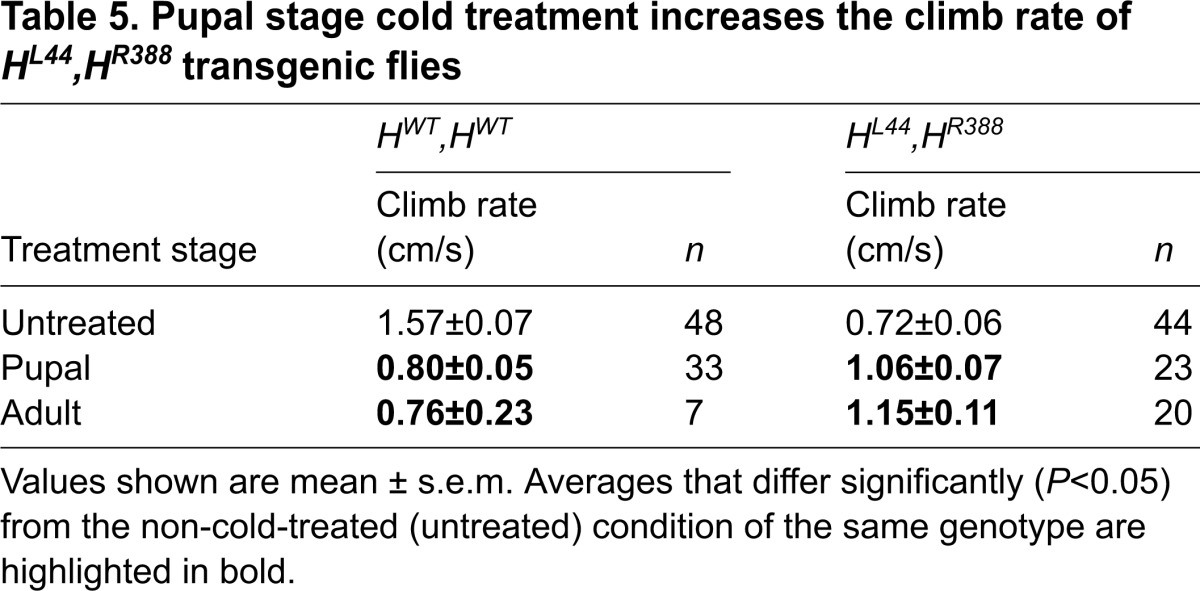
Pupal stage cold treatment increases the climb rate of *H^L44^,H^R388^* transgenic flies

### Cooling rescues synapse morphology defects in *spastin* larvae

**Fig. 4.**
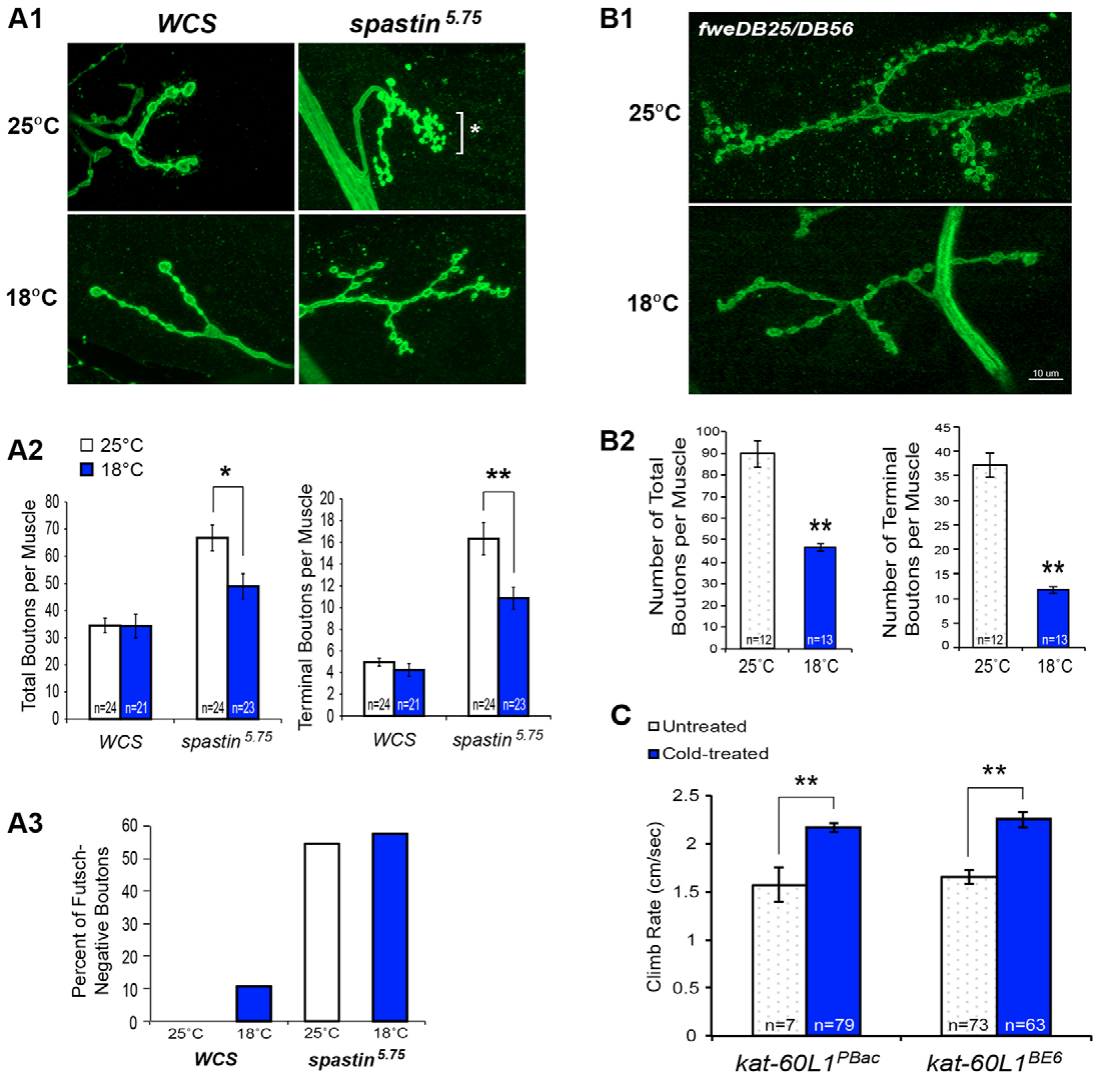
**Cooling rescues synapse morphology in both *spastin* and *flower* mutants, as well as climb rates in *kat-60L1* mutants.** (A1, A2) Synaptic terminals of *spastin^5.75^* larvae raised at 25°C have smaller, more numerous and bunched (*) boutons, unlike linearly arrayed *WCS* boutons. Rearing at 18°C restores mutant synapse morphology towards wild type, as measured by total number of synaptic boutons per muscle (*P*<0.02 compared with 25°C mutants) and terminal bouton number, a measure of arbor branching (*P*<5×10^−3^). Bunches are not observed in cold-treated larvae (*P*<0.03). (A3) Cooling does not rescue the distribution of stable microtubules within synaptic boutons, as seen by the increased number of boutons without Futsch immunostaining in *spastin^5.75^* mutants, even with cold treatment. (B1) Mutants in *flower*, which mediates synaptic vesicle endocytosis, also exhibit small, bunched synaptic boutons at the larval NMJ that are rescued to more wild-type morphologies by cooling. (B2) Both total and terminal bouton numbers are significantly reduced in *fwe* larvae reared at 18°C compared with 25°C (*P*<4×10^−7^). (C) Pupal stage cooling increases climb rate in flies lacking Kat-60L1, a microtubule-severing protein related to Spastin. Flies from cold-treated pupae expressing low (*kat-60L1^PBac^*) or no *kat-60L1* (*kat-60L1^BE6^*) climb faster than untreated flies of the same genotype (*P*<8×10^−4^ and *P*<1×10^−8^, respectively). *P<0.05; **P<0.005.

We next looked at whether cooling rescues the cell biology of neurons affected in *spastin* mutants. We were unable to address this issue in adults because the cellular loci of adult defects in *spastin* mutants remain unknown. We thus investigated *spastin* defects at the larval NMJ, which are well characterized (Sherwood et al., 2004). Although previous experiments showed that synaptic transmission in mutants is reduced relative to wild type at both normal and low temperatures, we examined whether other cellular defects at this stage could be alleviated by cold. At room temperature, *spastin* mutants have a distinctive NMJ morphology, with greater numbers of smaller synaptic boutons, which are sometimes arrayed in grape-like ‘bunches’ and contain only sparse microtubules (Fig. 4A) (Sherwood et al., 2004; Du et al., 2010; Ozdowski et al., 2011). These bunched terminal arrangements are not observed at wild-type NMJs, which consist of large, round and linearly arrayed boutons penetrated by a clear microtubule bundle. Comparison of synaptic terminals in 18°C-versus 24°C-reared larvae showed that cooling mitigated the *spastin* mutant morphology (Fig. 4A1,A2), both with respect to the total number of boutons per muscle and the number of terminal boutons (a measure of synaptic arbor branching). *WCS* synapse morphology was unaffected. However, we did not detect a change in stable microtubule penetration into terminal boutons, measured by the 22C10 antibody against the *Drosophila* MAP1b ortholog, Futsch (Fig. 4A3). Larval cooling thus partially rescues the cellular defects resulting from *spastin* loss, mitigating bouton morphology but not stable microtubule distribution or synaptic function.

### Mutants in *flower* and *kat-60L1* are also rescued by cold treatment

We have shown that cooling elicits effects specifically on *spastin* mutant phenotypes in comparison with controls. To help in understanding whether cold exerts a general rescuing effect on defective synaptic growth or whether these effects are also specific to mutations in *spastin*, we examined animals with mutations in *flower* (*fwe*), a putative Ca^2+^ channel that regulates synaptic vesicle endocytosis and has no known link to microtubule regulation (Yao et al., 2009). Similar to *spastin* mutants, however, *fwe^DB25/DB56^* larval NMJs have smaller and more numerous boutons, often arrayed in bunches, and are compromised in synaptic transmission. Rearing *fwe^DB25/DB56^* larvae at 18°C significantly reduced synaptic bouton number and arrangement to resemble wild-type morphologies (Fig. 4B), suggesting that the effects of cooling are not limited to mutations in *spastin* function.

We also examined adult behavioral phenotypes in mutants of a gene closely related to *spastin*, *kat-60L1*. Like Spastin, Kat-60L1 severs microtubules and is required during larval and pupal neuronal development, although the precise roles of each protein are distinct (Lee et al., 2009; Stewart et al., 2012). We examined the effect of cold on adult fly mobility in *kat-60L1^PBac^*, a partial loss of function line that acts as a strong hypomorph, and in *kat-60L1^BE6^*, a transcriptional null (Stewart et al., 2012). Both alleles are homozygous-viable and survive more successfully than *spastin^5.75^* null mutants, but also climb more slowly than *WCS* flies. Pupal cold treatment increased the average climb rates of both mutants over 35% relative to untreated mutant controls (Fig. 4C), to rates comparable to *WCS* controls (compare with Table 2).

## DISCUSSION

We have demonstrated that cold temperature alleviates reduced mobility and survival caused by loss of Spastin function in *Drosophila*. This is the case for flies lacking endogenous *spastin*, as well as those expressing pathogenic human Spastin. Cold treatment during the pupal stage of development was sufficient to enhance the eclosion rate, climbing ability and lifespan of *spastin* mutant adults. Furthermore, cold administered only after pupal development, to fully developed adults, also improved mutant mobility. The timing of these two effective periods is consistent with the idea that cold alleviates *spastin* mutant phenotypes by acting on the developing adult nervous system during pupal metamorphosis, but is also potent after the nervous system has matured. This is extremely promising from a clinical viewpoint, suggesting that the therapeutic window in AD-HSP includes both developing and mature nervous systems.

Although wild-type levels of mobility and survival were not often achieved, the temperature shift to 18°C conferred considerable improvement. Some cold-treated flies were able to jump and even fly briefly, behaviors not observed in untreated mutants. Cooling can match or exceed the efficacy of rescue by the microtubule destabilizing drug vinblastine, which has been proposed as a therapeutic approach for AD-HSP (Orso et al., 2005). Orso and colleagues showed that vinblastine doubled the ~12% eclosion rate of *spastin^5.75^* null mutants; in our hands the drug was ineffective for null and *H^L44^,H^R388^* eclosion, but improved eclosion by 65% for *H^WT^,H^R388^*, which is a more common, representative AD-HSP genotype associated with milder pathogenesis (Du et al., 2010; Fang Du and N.T.S., unpublished results). In comparison, pupal cooling of *spastin^5.75^* null mutants increased eclosion by 70%.

Importantly, cooling during the pupal and adult stages did not affect eclosion or motor behavior in wild-type flies. This suggests that cooling not only compensates for defects in neuronal function caused by lack of Spastin (or other mutations), but is also innocuous to properly functioning neurons. Although cooling administered at the larval stage was ultimately deleterious to both control and *spastin* mutant adults, mutant larval synapses were effectively restored to wild-type morphologies. This suggests that cold was beneficial for some *spastin*-mediated defects at this stage, but also had nonspecific, toxic effects on a cell population required later, in adults.

What is the mechanism(s) underlying the rescuing effect of cold? Our demonstration that cold alleviates not just *spastin* mutant phenotypes, but also mutant phenotypes in *fwe* and *kat-60L1*, indicates that that rescuing effects of cold on nervous system function might be quite broad. All three genes are important in synapse formation, although *kat-60L1* has been shown to act post-rather than pre-synaptically at larval and pupal stages. Reduced temperature could thus be generally beneficial to synaptic dysfunction, perhaps by reducing activity or metabolic load. Alternatively, *fwe*, *spastin* and *kat-60L1* might share a common pathway component(s), as yet undiscovered, that is directly affected by cold. For example, cold itself is well known to destabilize microtubules, particularly at temperatures below 20°C (Delphin et al., 2012), and is often used in experiments to depolymerize microtubules (Baas et al., 1994; Cottam et al., 2006). Cold could thus substitute directly for the microtubule-severing function of Spastin by promoting microtubule destabilization. Cold-mediated rescue of Kat-60L1 mutants supported this idea; however, we did not observe obvious differences in stable microtubule distribution at cold-treated *spastin^5.75^* synapses or in *Drosophila* S2R+ cells (data not shown), and *fwe* mutants, which have not been implicated in microtubule dysregulation, were also rescued by cooling.

In humans, cooling has been shown to be generally neuroprotective, and mild or moderate therapeutic hypothermia (e.g. 33–35°C) has long had clinical applications, including reducing neurological injury in patients following cardiac arrest, traumatic brain injury, epilepsy and stroke (Hartemink et al., 2004; Yenari and Han, 2012). Furthermore, Yang and colleagues found that exposure to even near-freezing temperatures results in minimal neuropathology in rat and cat neocortex and hippocampus (Yang et al., 2006). Although commonly administered in situations involving acute brain injury, the mechanism by which cooling confers neuroprotection or therapeutic improvement is unknown, multifactorial and context-dependent (Choi et al., 2012; Yenari and Han, 2012).

It will be important to characterize the *in vivo* effects of cold in mouse models of AD-HSP (Kasher et al., 2009; Fassier et al., 2013). The specificity of the effect of cold on mutant and not wild-type animals in our experiments, together with the spatially localized neurodegeneration in AD-HSP, suggest that moderate hypothermia could be applied in a highly targeted manner in this disease context, with minimal negative effects. Future studies should furthermore elucidate the underlying cellular mechanisms and potentially broader applications of cold in alleviating neuronal dysfunction in neurodegeneration. Because *Drosophila* are ectothermic, with body temperatures that vary with their environment, they provide a straightforward system in which the cell biological effects of temperature change can be studied *in vivo*. Finally, our data highlight the potential sensitivity of neuronal phenotypes to variations in temperature and thus its importance as a consideration in studies of neuronal function, neurodegeneration and behavior.

## MATERIALS AND METHODS

### *Drosophila* strains

Flies were reared on standard cornmeal/agar/molasses medium at either 24–25°C (untreated) or 18°C (cold treatment). Genetic controls were *white-CantonS* (*WCS*), the *CantonS* wild-type strain backcrossed to *white* ten times (gift of Anne Simon, Western University, Ontario, Canada) (Sherwood et al., 2004). Homozygous *spastin* null (*spastin^5.75^/spastin^5.75^*) animals were obtained by picking non-Tubby larvae from *spastin^5.75^/TM6B* stocks. Transgenic line *H^L44^,H^R388^* carried one copy each of transgenes encoding S44L and K388R human *Spastin* mutations, expressed via the inducible *geneswitch elav-GAL4* driver (Osterwalder et al., 2001), in the *spastin^5.75^* null background. *H^WT^,H^WT^* flies had two copies of wild-type human *Spastin* in the same background (Du et al., 2010). Kat-60L1 mutants were generated as described previously (Stewart et al., 2011). Trans-heterozygous *flower^DB25/DB56^* larvae were maintained at 25°C or 18°C after collecting non-green progeny at ~stage L2, of *fwe^DB25^*/TM3,Ser,twi-GAL4,UAS-GFP × *fwe^DB56^*/TM3,Ser,twi-GAL4,UAS-GFP adults allowed to lay on yeasted grape juice plates.

### Assessment of cold treatment on behavioral phenotypes

Third-instar larvae of the appropriate genotype were picked with a cotton swab and placed in a clean vial. Eclosion rate was calculated as the number of adult flies from each vial divided by the number of larvae originally placed into that vial. After eclosing, each adult fly was moved to an individual food vial.

Adult mobility was assessed via a climb rate test: single flies were transferred to an empty vial, tapped to the bottom, and the rates at which they climbed up to an 8-cm mark measured. This was repeated three times per fly and the results averaged. Flies were then transferred back into their individual food vial and replaced at either 24°C or 18°C depending on experimental group. All tests were done at 24°C; adults maintained at 18°C were first equilibrated for 30 minutes at 24°C prior to being transferred to the empty vial for testing. Mobility was measured using flies aged 0–27 days. The average age of flies tested was not correlated with the average climb rates.

Adults were transferred to new food vials every 1–2 weeks to maintain healthy living conditions. Lifespan was the number of days from eclosion until death.

The Student’s *t*-test was used to measure statistical significance. Graphs are presented as percentage changes due to the large differences between wild-type and *spastin* mutant controls; raw values (mean ± s.e.m.) and number (*n*) of experiments are listed in the tables and figures.

### Neuromuscular junction immunohistochemistry

Third-instar *WCS* and homozygous *spastin^5.75^* or trans-heterozygous *flower^DB25/DB56^* larvae were filleted, dissected and immunostained using standard methods (e.g. Ozdowski et al., 2011). Briefly, larvae were dissected in room temperature PBS and fixed for 30 minutes in 4% paraformaldehyde, immunostained at 4°C overnight using the neuronal membrane marker rabbit anti-HRP (1:100; Jackson ImmunoResearch, PA, 323-005-021) alone or with mAb 22C10 to label microtubules (mouse anti-Futsch, 1:50; Developmental Studies Hybridoma Bank, University of Iowa, Iowa City, IA). Secondary antibodies (Alexa Fluor 488 goat anti-rabbit A-11070 and Alexa Fluor 568 goat anti-mouse A-11031; 1:400; Life Technologies, Grand Island, NY) were incubated for 2–3 hours at room temperature. Fillets were mounted in H-1000 (Vector Laboratories, Burlingame, CA) and z-series images of muscle 4 synapses from larval segments 2–4 acquired on a Zeiss LSM 510 inverted confocal microscope using 63× 1.4 N.A. or 100× 1.2 N.A. PlanApo objectives (Oberkochen, Germany).
